# Jasmonates: Multifunctional Roles in Stress Tolerance

**DOI:** 10.3389/fpls.2016.00813

**Published:** 2016-06-15

**Authors:** Parvaiz Ahmad, Saiema Rasool, Alvina Gul, Subzar A. Sheikh, Nudrat A. Akram, Muhammad Ashraf, A. M. Kazi, Salih Gucel

**Affiliations:** ^1^Department of Botany, S.P. CollegeSrinagar, India; ^2^Department of Botany and Microbiology, College of Sciences, King Saud UniversityRiyadh, Saudi Arabia; ^3^Forest Biotech Lab, Department of Forest Management, Faculty of Forestry, Universiti Putra MalaysiaSelangor, Malaysia; ^4^Atta-ur-Rahman School of Applied Biosciences, National University of Science and TechnologyIslamabad, Pakistan; ^5^Department of Botany, Govt. Degree College (Boys), AnantnagAnantnag, India; ^6^Department of Botany, GC University FaisalabadFaisalabad, Pakistan; ^7^Pakistan Science FoundationIslamabad, Pakistan; ^8^Department of Botany, University of SargodhaSargodha, Pakistan; ^9^Centre for Environmental Research, Near East UniversityNicosia, Cyprus

**Keywords:** jasmonate biosynthesis, MAPK cascades, physiological responses, plant stress tolerance, signaling pathway

## Abstract

Jasmonates (JAs) [Jasmonic acid (JA) and methyl jasmonates (MeJAs)] are known to take part in various physiological processes. Exogenous application of JAs so far tested on different plants under abiotic stresses particularly salinity, drought, and temperature (low/high) conditions have proved effective in improving plant stress tolerance. However, its extent of effectiveness entirely depends on the type of plant species tested or its concentration. The effects of introgression or silencing of different JA- and Me-JA-related genes have been summarized in this review, which have shown a substantial role in improving crop yield and quality in different plants under stress or non-stress conditions. Regulation of JAs synthesis is impaired in stressed as well as unstressed plant cells/tissues, which is believed to be associated with a variety of metabolic events including signal transduction. Although, mitogen activated protein kinases (MAPKs) are important components of JA signaling and biosynthesis pathways, nitric oxide, ROS, calcium, ABA, ethylene, and salicylic acid are also important mediators of plant growth and development during JA signal transduction and synthesis. The exploration of other signaling molecules can be beneficial to examine the details of underlying molecular mechanisms of JA signal transduction. Much work is to be done in near future to find the proper answers of the questions like action of JA related metabolites, and identification of universal JA receptors etc. Complete signaling pathways involving MAPKs, CDPK, TGA, SIPK, WIPK, and WRKY transcription factors are yet to be investigated to understand the complete mechanism of action of JAs.

## Introduction

Plants being sessile can respond to environmental cues through a variety of physio-biochemical processes and structural modifications (Ahmad et al., 2008; Farrant and Ruelland, 2015). Plants produce volatile and non-volatile compounds including phytohormones that help them to adapt to changing environment (Bari and Jones, 2009; Ashraf et al., 2010; Javid et al., 2011). Phytohormones have a leading role in various physiological and developmental processes in plants (Rohwer and Erwin, 2008; Ashraf et al., 2010; Kumar et al., 2014). An important phytohormone, JA (jasmonic acid) and its methyl ester, methyl jasmonates (MeJAs), are derivatives of the fatty acid metabolism (Kupper et al., 2009; Gao et al., 2011; Jalalpour et al., 2014). JA is ubiquitously found in the plant kingdom (Schaller, 2011; Pirbalouti et al., 2014). Jasmine (*Jasminum grandiflorum*) oil is used as a primary source to isolate MeJA (Avanci et al., 2010). JA was isolated for the first time from the culture of fungus *Lasiodiplodia theobromae* which is an important member of the JAs (Tsukada et al., 2010). Apart from JA and MeJA, other JAs particularly *cis*-jasmone, jasmonoyl ACC (JA-ACC), and jasmonoyl isoleucine (JA-Ile) with multiple biological functions have been reported (Rohwer and Erwin, 2008; Avanci et al., 2010; Wasternack and Kombrink, 2010; Koo and Howe, 2012). Tuberonic acid (TA) and its glucoside (TAG) were found in the leaves of *Solanum tuberosum* (Wakuta et al., 2010; Seto et al., 2011), which were believed to be involved in tuber formation. In various plant species, these compounds can be biosynthesized by hydroxylation and subsequent glycosylation (Wakuta et al., 2010). Cucurbic acid, a compound similar to JA, identified in the seeds of *Cucurbita pepo* (Nam and Yoshihara, 2008), was reported to be actively involved in tuberization (Honda et al., 2006). JA and MeJA analogs with physiological activities have also been reported (Kazan and Manners, 2008, 2011; Baenas et al., 2014). For example, Kondo et al. (2000) reported an increase in the ABA and anthocyanin contents in apples through PDG (*N*-propyl dihydrojasmonate). Coronatine isolated from *Pseudomonas syringae* is a phytotoxin and is the most active isomer of JA. It has been reported to be involved in alkaloid accumulation in *Eschscholzia californica*, tendril coiling in *Bryonia dioica*, and tuber cell expansion in *Solanum tuberosum* (Koda, 1997; Rohwer and Erwin, 2008).

Jasmonate and MeJA are believed to play an active role in senescence. In addition, a variety of JA-induced plant growth, developmental and physiological activities have been reported including fertility, biotic and abiotic stress tolerance, sex determination, storage organ formation, reproductive processes, root elongation, fruit ripening and senescence, oxidative defense, and interaction with other hormones (Browse, 2009; Moreno et al., 2009; Avanci et al., 2010; Cipollini, 2010; Nafie et al., 2011). Other physiological functions related to the JA are stimulation of germination in dormant seeds (Creelman and Mullet, 1997), accumulation of storage proteins (Pelacho and Mingo-Castel, 1991), chlorosis (Creelman and Mullet, 1997), upregulation of antioxidant enzymes (Soares et al., 2010), senescence (Seltmann et al., 2010), floral nectar synthesis (Radhika et al., 2010a,b), herbivory and wounding (Chung et al., 2008; Howe and Jander, 2008; Ballare, 2011; Erb et al., 2012), seed and flower development (Wasternack et al., 2012), systemic resistance (Pieterse et al., 2002, 2012), elicitors of plant secondary metabolism (De Geyter et al., 2012), and allelopathy (Baldwin, 2010), etc. Its role in gene expression has been reported in different plants such as grapevines (Marchive et al., 2013), *Arabidopsis* (Sasaki et al., 2001), tomato (Boter et al., 2004), rice (Schweizer et al., 1997; Liu et al., 2012), sugarcane (De Rosa et al., 2005), etc., which leads to defense against environmental stresses (Gfeller et al., 2010b; Ballare, 2011; Sua et al., 2011).

In plants, the concentration of JAs ranges from 0.01 to 3.0 ng/g FW (fresh weight) with the exception of *Artemisia tridentata* in which MeJA level up to 95 μg g^-1^ fresh weight has been recorded (Preston et al., 2004). JA has been found in abundance generally in flowers and chloroplasts of illuminated plants (Creelman and Mullet, 1997; Yan et al., 2013).

Research on JA signaling is gaining ground day by day, because of the primary reason that they are involved in regulation of tolerance against different environmental stresses (Fonseca et al., 2009; Koo et al., 2009; Gfeller et al., 2010a; Wasternack and Hause, 2013) as well as their integration with the signaling pathways generated by salicylic acid, ABA, ethylene, and other such molecules. In fact, most of the information on JA signaling has been derived from studies on two widely researched plants *Arabidopsis* and tomato (Turner et al., 2002; Browse and Howe, 2008). One could expect considerable variation among JA signaling pathways operative in other species with complex genomes exposed to different stresses. Thus, the present review focuses on elucidation of the role of JAs in various physiological and molecular processes involved in stress tolerance, as well as it offers important details about the signaling pathways in plant stress tolerance mechanisms.

## Jasmonate Biosynthesis

**FIGURE 1:**
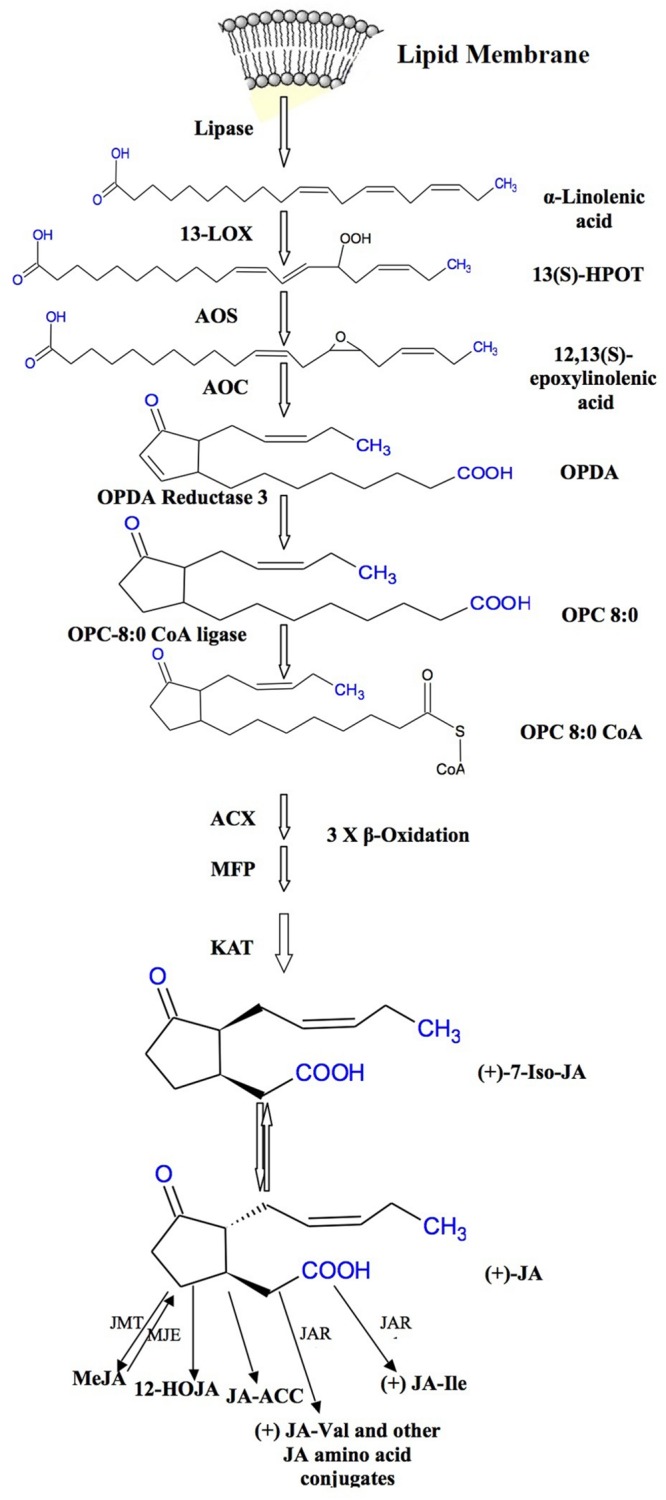
**Biosynthetic pathway of jasmonates**.

Jasmonic acid biosynthetic pathway was first described by Vick and Zimmerman (1984), and since then it has been thoroughly studied in *Arabidopsis* and tomato. JAs and MeJA are produced from α-linolinic acid (α-LeA) localized in chloroplast membranes (Wasternack and Kombrink, 2010). The formation of α-LeA from lipids occurs through the action of phospholipases. An intermediate compound 13-hydroperoxy-9, 11, 15-octadecatrienoic acid (13-HPOT) is formed by the addition of oxygen molecule to α-LeA and the reaction is stimulated by 13-lipoxygenase (LOX). This 13-HPOT is then oxidized to allene oxide by allene oxide synthase (AOS). Allene oxide is an unstable compound which is then converted to 12-oxo phytodienoic acid (12-OPDA) by the enzyme allene oxide cyclase (AOC). The AOS and AOC are present in plastids and they act in concert (Schaller and Stintzi, 2009; Gfeller et al., 2010b; Wasternack and Hause, 2013). The enzyme AOS belongs to the cytochrome P450 family, a potential catalyst for JA biosynthesis (Schaller and Stintzi, 2009). The final product of metabolic pathway of JA biosynthesis is 12-OPDA which occurs in the chloroplast which undergoes three cycles of β-oxidation in the peroxisomes (Delker et al., 2006; Wasternack, 2007; Bussell et al., 2013). **Figure 1** clearly shows the JA formation from 12-OPDA with different metabolic conversions forming distinct JAs. MeJA is formed by the methylation reaction in the presence of JA methyltransferase. It has been reported that AOC is encoded by one gene in tomato but by a gene family (small) in *Arabidopsis* (Wasternack and Hause, 2013). Some cDNA studies have shown that AOS is a protein having C-terminal domain identical to cytochrome P450s containing heme-binding cysteine and is composed of about 536 amino acids (Schaller and Stintzi, 2009). It is not clear as to how 12OPDA enters in peroxisomes, wherein final steps of JA synthesis take place. However, some complex and contrasting reports on this process are available. Thus, it still needs to be elucidated that how the JAs production is regulated initially and its synthesis is limited particularly in stressed plant cells/tissues.

## Exogenous Application of Jasmonates and Plant Stress Tolerance

**FIGURE 2:**
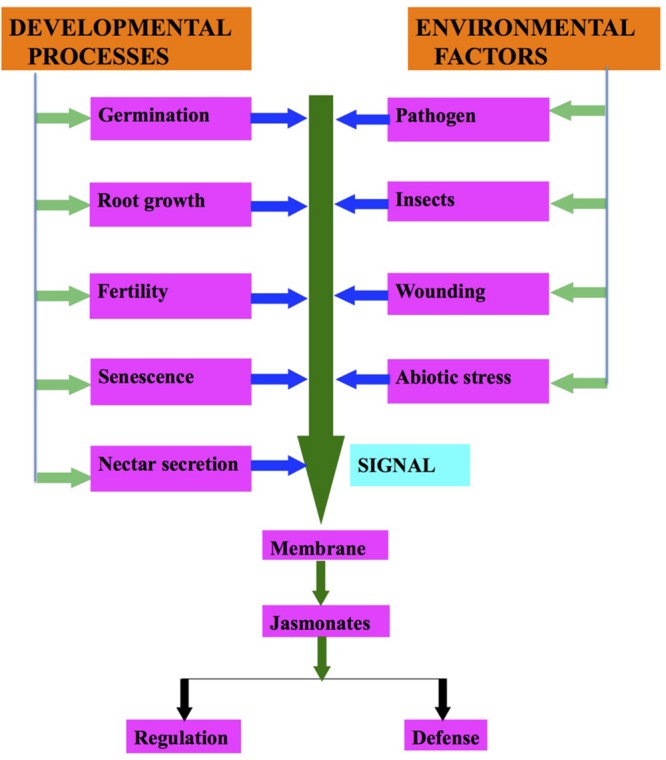
**Regulation of jasmonates biosynthesis due to developmental processes and environmental factors**.

Under environmental stresses (biotic and abiotic), plants show a wide range of responses from synthesis of signaling compounds to eventual cell death (Avanci et al., 2010; **Table 1**; **Figure 2**). Environmental stresses induce the synthesis of different plant hormones (**Figure 2**). These phytohormones are required at various developmental stages of the plants and help in their defense responses (Ashraf et al., 2010; Denancé et al., 2013). Rosahl and Feussner (2005) demonstrated that JAs and their derivatives regulate the gene expression involved in defense responses. JA dependent defense responses are activated as a result of necrotrophic pathogen infection (Trusov et al., 2006; Avanci et al., 2010).

**Table 1:** Improvement in different physio-biochemical attributes of different crop plants by exogenous application of different types of jasmonates.

Type of stress	Compound used	Concentration	Mode of application	Crop	Characteristics improved	Reference
Drought stress	Methyl jasmonate (MeJA)	0.5 mM	Foliar spray	Spearmint (*Mentha spicata* L.)	Concentration of beta-caryophyllene increased	Zheljazkov et al., 2013
Drought stress	MeJA	0.2, 0.5, and 1.0 mmol L^-1^	Foliar spray	Tobacco (*Nicotiana tobaccum* L.)	Showed positive effects by improving *F_v_/F_m_, F*v/*F*o, ETR, qP, and qN under drought stress. Foliar applied MeJA alleviated degradation of chlorophyll and played a critical role in protecting PSII under drought stress	Wei-Wei et al., 2011
Control (non-stress conditions)	MeJA	0.5, 1.0, and 2.0 mM	Foliar spray	Pomegranate (*Punica granatum* L.)	Phenolic compounds and antioxidant activity of pomegranate fruit increased	Vatanparast et al., 2012
Fungal infection	MeJA	100 mM	Seed pretreatment	Norway spruce [*Picea abies* (L.) Karst.]	Pathogen infection, mechanical wounding, and bark beetle attack minimized	Krokene et al., 2008
No stress	MeJA	0.25, 0.5, and 0.75 mmol L^-1^	Foliar spray	Tomato (*Lycopersicon esculentum*)	MeJA improved vegetative and reproductive growth, yield and chlorophyll content of tomato plants, while had no significant effect on blossom end rot and leaf N, K content	Kazemi, 2014
No stress	MeJA	100 mmol L^-1^	Foliar spray	*Picea abies*	It induced swelling of existing polyphenolic parenchyma (PP) cells and increased their phenolic contents and formation of additional PP cells	Franceschi et al., 2002
Salt stress	MeJA	0.1 and 0.01 μM	Seed soaking	*Ocimum basilicum* L.	MeJA improved seed germination percentage and stress tolerance in plants	Enteshari and Jafari, 2013
Salt stress	MeJA	5 mM	Foliar spray	Broccoli (*Brassica oleracea* L.	MeJA maintained growth, gas exchange parameters, and leaf N-NO_3_ levels, while reduced Na^+^ concentration at low saline level. However, at a higher salt concentration i.e., 120 mM NaCl, no significant effect of MeJA was observed	del Amor and Cuadra-Crespo, 2011
Drought stress	MeJA	0.25 μM	Foliar spray	Wheat (*Triticum aestivum* L.)	MeJA enhanced drought tolerance by increasing dark respiration rate, photosynthesis and the activities of SOD, POD, CAT enzymes, delayed plant senescence, and reduced MDA content mainly by improving the water status of wheat plants	Ma et al., 2014
Salt stress	MeJA	20 and 30 μM	Rooting medium	Soybean (*Glycine max* L.)	It improved plant growth, leaf photosynthetic and transpiration rate, chlorophyll and proline contents	Yoon et al., 2009
Drought stress	Jasmonic acid (JA)	50 mmol m^-3^	Foliar spray	Pear	Enhanced betaine level, BADH activities and BADH protein contents	Gao et al., 2004
Drought stress	MeJA	50 μM	Foliar spray	Soybean	Improved drought tolerance in soybean plants by decreasing membrane lipid peroxidation and increasing antioxidant activities	Anjum et al., 2011
No stress	MeJA	0.2 and 0.4 mM	Foliar spray	Rhodes grass (*Chloris gayana* Kunth.)	Exogenous MeJA significantly increased the densities of macro-hairs and salt glands on the adaxial and abaxial leaf surfaces and those of prickles on the adaxial leaf surface	Kobayashi et al., 2010
No stress	MeJA	1120, 2240, and 4480 mg L^-1^	Foliar spray	Apple	Improved ethylene, anthocyanin, and phenolic content, and antioxidant capacity increased linearly with increasing MeJA concentrations, regardless of the application interval: although MeJA treatments increased ethylene biosynthesis, they did not cause any softening; on the other hand, fruit firmness increased linearly with increasing MeJA concentrations	Ozturk et al., 2014
Cadmium stress	MeJA	0.1–1 μmol L^-1^	Rooting medium	*Kandelia obovata*	Improved ascorbic acid contents and the activities of CAT and APX in *Kandelia obovata*, resulted in decreased Cd-induced oxidative damage	Chen et al., 2014
Chilling stress	MejA	10^-4^ M	Foliar spray	Mango	Exogenous application of MeJA improved fruit quality and total soluble solids, and enhanced chilling tolerance by reducing ion leakage in mango tissue	Gonzalez-Aguilar et al., 2000
No stress	JA	0.5, 5.0, and 10 μM	Foliar spray	Melon (*Cucumis melo* L.)	Regulated primary as well secondary metabolism, and enhanced antioxidant enzyme activities and contents of ascorbic acid, coumarin and *p*-coumaric contents	Nafie et al., 2011
No stress	MeJA	250 μM	Foliar spray	Kale	MeJA significantly accelerated glucobrassicin (98%), quinone reductase, gluconasturtiin (56%), and neoglucobrassicin (150%) contents in the leaf of kale plants	Ku et al., 2014
No stress	MeJA	300 μM	Foliar spray	*Artemisia annua*	MeJA increased artemisinin content, but no correlation was found between gene expression and its content. MeJA-induced increase in artemisinin content may have been due to some other mechanisms	Mehrjerdi et al., 2013
No stress	MeJA	22.4 μL L^-1^	Foliar spray	Raspberry (*Rubus idaeus* L.)	MeJA treated plants showed highest antioxidant capacity measured in terms of oxygen radical absorbance capacity. In addition, improved activities of SOD, guaiacol peroxidase, ascorbate peroxidase, glutathione peroxidase, glutathione reductase, monodehydroascorbate reductase, and dehydroascorbate reductase enzymes along with contents of AsA, dehydroascorbate, reduced and glutathione oxidized	Chanjirakul et al., 2006
No stress	MeJA	8, 16, and 24 μL L^-1^	fumigation	Raspberry	Improved antioxidant capacity and total anthocyanins compared to the non-treated one but raspberry fruits could not maintain fruit quality	Ghasemnezhad and Javaherdashti,2008

Jasmonate treated plants have been found to show enhanced resistance against pests. Tomato plants treated with JA have shown reduced number of *Frankliniella occidentalis* (thrips), flea beetles and aphids, which were ascribed to an increase in the activities of polyphenol oxidase and proteinase inhibitors (Thaler et al., 2001). The expression of defense-related enzymes mainly lipoxigenase and peroxidase followed by polyphenol oxidase were significantly higher in plants damaged by ventral eversible gland intact (VEGI) caterpillars than that in plants damaged by ventral eversible gland ablated (VEGA) caterpillars. They observed that the genes encoding these enzymes were also involved in the biosynthesis of JA and terpene synthase genes, and subsequently, involved in the regulation of terpenes (Zebelo et al., 2014). JAs are also involved in the resistance to root pests like fungus gnat (*Bradysia impatiens*) as studied in *Arabidopsis* (McConn et al., 1997) and spinach (Schmelz et al., 2002). They suggested that the mutant will be a good genetic model for testing the practical effectiveness of defensive genes as they observed that exogenous application of MeJA significantly reduced mortality to ≈12% and protected the mutant plants. Thus, plants are equipped with sophisticated defense systems to protect themselves against the attack of herbivorous organisms (Ballare, 2011).

It has been observed that the sorbitol treated barley leaves showed enhancement in JA and JA-induced protein levels (Rohwer and Erwin, 2008), proving the role of JAs in osmotic stress. JAs are found to regulate the stomatal apertures (Hossain et al., 2011; Riemann et al., 2015). Exogenous JA (50 mmol m^-3^) application causes an increase in glycine betaine levels, activity of BADH enzyme as well as level of BADH protein in grafted *Pyrus bretschneideri* Redh subjected to water deficit conditions (Gao et al., 2004).

Reactive oxygen species (ROS) are very harmful for the growth and development of most organisms as these can affect the structure and function of biomolecules (Ahmad et al., 2008, 2010, 2011a,b,c; Koyro et al., 2011; Akram et al., 2012; Akram and Ashraf, 2013; Shafiq et al., 2014). The ROS include H_2_O_2,_ O_2_^-^, and ^∙^OH (Faurie et al., 2009; Akram et al., 2012). Soares et al. (2010) have demonstrated a gradual accumulation of H_2_O_2_ in *Ricinus communis* between 1 and 6 h after treatment with MeJA. They also observed a sharp formation of ROS at the initial moment of MeJA application and ascribed it to the decrease in the activities of enzymatic antioxidants. In another study with pomegranate (*Punica granatum* L.), Vatanparast et al. (2012) observed an increase in phenolic compounds and antioxidant activity in the foliage treated with 0.5–2.0 mM MeJA. Orvar et al. (1997) demonstrated that pre-treatment of tobacco plants with JA inhibited ozone induced cell death. They found that after 3–4 h of MeJA application the activity of ascorbate peroxidase was upregulated while that of carbonic anhydrase mRNA decreased in tobacco plants subjected to ozone treatments. JA reduced the amount of SA produced in response to O_3_ in *Arabidopsis* (Rao et al., 2000). MeJA reduced the adverse effects of drought and oxidative stresses in strawberry (Wang, 1999). Pre-treatment of barley seeds with MeJA showed less membrane damage than did the non-treated plants (Bandurska and Stroiński, 2003).

Methyl jasmonate application enhanced the amount of ascorbic acid in *Arabidopsis* and tobacco suspension cells (Wolucka et al., 2005) and influences the metabolism of AsA (Maksymiec and Krupa, 2002; Wolucka et al., 2005). AsA is a very important antioxidant, which protects the plants under oxidative stress (Ahmad et al., 2011a,b,c; Shafiq et al., 2014). It has been reported that exogenous application of JA increases AsA in different plants suggesting that JA may regulate AsA metabolism (Ayala-Zavala et al., 2008; Chen et al., 2014). Grantz et al. (1995) reported that AsA increased due to wounding and it led to improved stress resistance. Likewise, Suza et al. (2010) also demonstrated enhanced accumulation of AsA *Arabidopsis* by wounding.

In **Table 1**, it has been summarized that how exogenous application of JAs regulates a number of processes in different plants on exposure to different environmental stresses. JA has been reported to protect Zucchini from chilling injury (Wang and Buta, 1994). Exogenous application of MeJA improved fruit quality and enhanced chilling stress tolerance of mango (*Mangifera indica*) fruit without affecting fruit ripening process (Gonzalez-Aguilar et al., 2000). Droby et al. (1999) have reported that application of MeJA suppresses the fungal growth in grapefruit. Moreover, MeJA maintained the post-harvest quality of papayas (Gonzalez-Aguilar et al., 2003). MeJA enhanced the antioxidant system and the free radical scavenging capability of raspberry fruits plants making them more resistant to decay (Ghasemnezhad and Javaherdashti, 2008; Wang et al., 2009). External application of JAs also improves fruit quality, which is believed to be associated with a variety of metabolic events including signal transduction. Nitric oxide, ROS, calcium, ABA, ethylene, and salicylic acid are also important mediators of plant growth and development during JA signal transduction and synthesis.

## Jamonates and Physiological Responses

The first physiological effect demonstrated for JA was root growth inhibition (Adams and Turner, 2010; Liu et al., 2010). Coi1, Jin1, or Jar1 are JA-insensitive mutants and did not show any effect on root length during JA treatment and the root length was similar as compared to untreated wild-type (WT). JA-sensitive mutants like *Cex1, Cet1, Cev1*, and *Joe2* were characterized by stunted growth and suppressed root length. They exhibit overexpression of JA-induced genes (Pauwels et al., 2009).

Jasmonate and MeJA stimulated the germination of dormant seeds (Norastehnia et al., 2007; Dave et al., 2011) and it was observed that increase in JAs causes lipid peroxidation, which in turn causes membrane damage, hence resulting in enhanced germination (Ranjan and Lewak, 1992). In soybean plant, organs like hypocotyl hook, axes, and plumules showed higher levels of JA as compared to the hypocotyl zone of elongated cells and the non-elongating roots and stems (Creelman and Mullet, 1995). JA was first seen in Solanaceae species and recently in *Arabidopsis thaliana* (Gidda et al., 2003). In *A. thaliana* it is recognized as a substrate for *AtST2a* (sulfotransferase). AtST2a helps reduce the endogenous levels of 12-OH-JA, a hydroxylated product of JA (Gidda et al., 2003; Faraz, 2006; reviewed by Wasternack, 2007).

Staswick (1994) demonstrated that JA regulates the genes of vegetative storage proteins (VSPs). VSPs have been characterized and purified for the first time from soybean plant (Wittenbach, 1983). VSPs localize themselves in vacuoles and bundle sheath cells of soybean (Franceschi et al., 1983). Staswick (1989) demonstrated that accumulation of VSPs occurs in pods and developing reproductive parts excluding seeds, so it can be suggested that the VSPs are temporary deposits of amino acids obtained from disassembly of leaf proteins and Rubisco, that become active for seed formation. JA along with sugars, phosphate, nitrogen and auxin regulate these proteins (Creelman and Mullet, 1997). *A. thaliana AtVSPs* and soybean *VSPs* show high levels of expression in developing fruit as well as flowers (Bell et al., 1995). In *Coi1* mutant (insensitive to JA) of *A. thaliana*, the AtVSP proteins in flowers were absent and can be induced upon JA treatment (Benedetti et al., 1995). Deficient in JA *Arabidopsis* mutants like *dad1* and *opr3* showed reduced filament elongation associated with low accumulation of JA in the filaments of the double mutant *arf6/arf8*. These mutants are unable to produce two ARFs (auxin response factors) involved in filament elongation, which showed that auxin signaling is interlinked with JA (Wasternack, 2007).

Nodule is a bulbous root structure found in leguminous plants. It contains a bacterial enzyme nitrogenase, which is responsible for the fixation of atmospheric nitrogen. According to Stougaard (2000) rhizobial bacteria release lipochitooligosaccharide NOD factors, which have a role in nodulation initiation. Initiation of nodule development is dependent on signaling pathway of cytokinins (Tirichine et al., 2007; Reid et al., 2016). Many reports have shown that hormones like auxins, gibberellins, abscisic acid, ethylene, brassinosteroids etc. are involved in nodule development (Ferguson et al., 2005). Nodulation is regulated negatively by ethylene through the inhibition of Ca spiking due to Nod factor (Oldroyd and Downie, 2006; Ding et al., 2008). However, it is still unclear how nodule organogenesis and senescence are regulated by hormones. Nodulation inhibition by JA has recently been reported in *Medicago truncatula* (Sun et al., 2006) and *Lotus japonicus* (Nakagawa and Kawaguchi, 2006). EST analysis of *Lotus japonica* showed that during *Rhizobium*–*Lotus* interaction, genes involved in pathogen defense response and JA biosynthetic enzyme (AOC and OPRs) were upregulated and suppressed during the late nodule formation step (Shigeyama et al., 2012; Bordenave et al., 2013). According to Kouchi et al. (2004), in the JA biosynthetic pathway, genes responsible for lipooxygenase were mostly downregulated.

Senescence is one of the first physiological responses that were demonstrated by Ueda and Kato (1980) in *Avena sativa* (**Figure 2**). After that many authors have reported that JAs induce senescence (Schommer et al., 2008; Reinbothe et al., 2009). He et al. (2002) observed that an increase in JA levels activates the enzymes of JA biosynthetic pathway, which in turn activates the *SENESCENCE ASSOCIATED GENES* (*SAGs*). JA up-regulates the senescence related genes, which are: *SAG12, SAG14, SAG15, SEN1, SEN4, SEN5* (He et al., 2002). Expression of AOS and OPR3 in JA biosynthetic pathway and increase in JA are the indications of senescence due to JAs (van der Graaff et al., 2006). Seltmann et al. (2010) demonstrated that plants exhibit different phenotypes during senescence either induced or natural. However, they share symptoms like yellowing, etc.

In addition to organ senescence, JA plays an effective role in cell death as well as it reduces cell proliferation in human cancerous cells. However, no JAs have been reported from animals till date. Recently, it was reported that JAs also play a role in physiological response of secretion of floral nectar (**Figure 2**). Radhika et al. (2010a) demonstrated that floral nectar secretion is controlled by JAs in *Brassica* species. Interestingly, a significant production of floral nectar was observed in the flowers of *B. napus*, when JA is exogenously sprayed to them. However, nectar secretion decreased with decrease in JA application. JA also regulates nectar secretion in flowers as it causes secretion of defensive extra floral nectar (Bender et al., 2012; Heil, 2015). Yamane et al. (1982) demonstrated the occurrence of MeJA and JA in pollens and/or anthers of *Camellia* spp. On the background of *Arabidopsis* mutants it has been proposed that JAs are important for pollen development, stamen elongation and the pollen release timing (Liechti and Farmer, 2006). The dehiscence of anthers is delayed in the *delayed dehiscence 1* (*dde1*) mutant of *Arabidopsis* resulting in inefficient fertilization. JA treatment has been found to restore the WT phenotype and helps the plant to produce seeds. During flower maturation, DDE1 accumulated in pistil, petals, and anther filamental tissues, but not in stomium. These results lead to the conclusion that JA-signaling regulates anther dehiscence. Studies on *A. thaliana* suggest that JA is capable of inducing and coordinating anther filament elongation, stomium opening at anthesis, and for viable pollen production (Stintzi and Browse, 2000). Mandaokar et al. (2006) have demonstrated that 13 transcription factors (TFs) such as MYB21 and MYB24, are involved in stamen maturation. These TFs are induced by JA, and they stimulated stamen development (Avanci et al., 2010). However, it is not yet explicit how endogenous levels of different components are altered due to its application. This certainly needs to be researched.

## Jasmonate Signaling Pathway

Jasmonates are synthesized from lipid constituents and perceived by protein receptors that activate signal transduction pathway (Wasternack and Hause, 2013). This pathway brings modulations in plant responses regulated by JAs from transcription to translation (Wasternack and Hause, 2002; Santino et al., 2013; Wasternack and Hause, 2013). JAs are responsible for the inhibition of root length and thus have been used for exploring JA signaling mutants. The pioneer JA deficient mutant observed in *Arabidopsis* was *Coronatine-insensitive1* (*Coi1*; Kazan and Manners, 2008). Reduced sensitivity in root elongation has been shown by *Coi1* mutants when treated with JA and coronatine (COR). COR, a phytotoxin secreted by *P. syringae* (a bacterial pathogen) is structurally and functionally similar to JA. The *Coi1* mutants are defective in various functions that are JA dependent, e.g., pest resistance, pathogen resistance, wound response, secondary metabolite biosynthesis and fertility. The *Coi1* gene codes for a protein having 16 leucine-rich repeats and F-box motif (Browse, 2009; Avanci et al., 2010). F-box protein forms multiprotein structures that serve as receptors (Avanci et al., 2010). According to Xie et al. (1998) *Coi1* participated in removing repressors of the JA transduction. These repressors are mediated by SCF complexes found in all eukaryotes (Jiang et al., 2013; Wasternack and Hause, 2013). The composition of SCF complex is of proteineous type and is made up of SKP1, F-box and Cullin proteins. The SCF complex controls cell cycle regulation by activating ubiquitination of the cell cycle proteins and their degradation by the 26S proteasome (Kazan and Manners, 2008).

Analysis of the *Arabidopsis jar1* and *jai1* mutants revealed that JAI (JASMONATE INSENSITIVE1) and JAR1 (*JASMONATE RESISTANT1*) have been reported to suppress effectiveness of externally applied JAs. Jar1 mutants are highly sensitive to pathogens (Rosahl and Feussner, 2005). *JAR1* encodes a JA amino acid synthetase, and facilitates JA link to isoleucine thereby showing its role as a signaling molecule by suppressing root growth (Staswick, 2008; Wasternack and Kombrink, 2010). Staswick and Tiryaki (2004) demonstrated that AMP is replaced with an amino acid in JAR1. JAR4 a homolog of JAR1 have been recently cloned from *Nicotiana attenuata* (Kang et al., 2006). JAR4 deficient plants are more sensitive to insect *Manduca sexta*, demonstrating the importance of JA-Ile in plant defense (Wasternack, 2007).

JASMONATE INSENSITIVE1 (Jai1) is a sterile mutant of tomato and is resistant to JA (Li et al., 2004). Jai1 is a tomato coi1 homolog as Jai showed 68% amino acid identity with coi1 (Li et al., 2004). Jai1 mutant plants showed loss of seed maturation control, glandular trichomes appearance on leaves, unripened fruits and sepals indicating JA-signaling pathway in glandular trichomes of tomato defense responses (Li et al., 2004).

**FIGURE 3:**
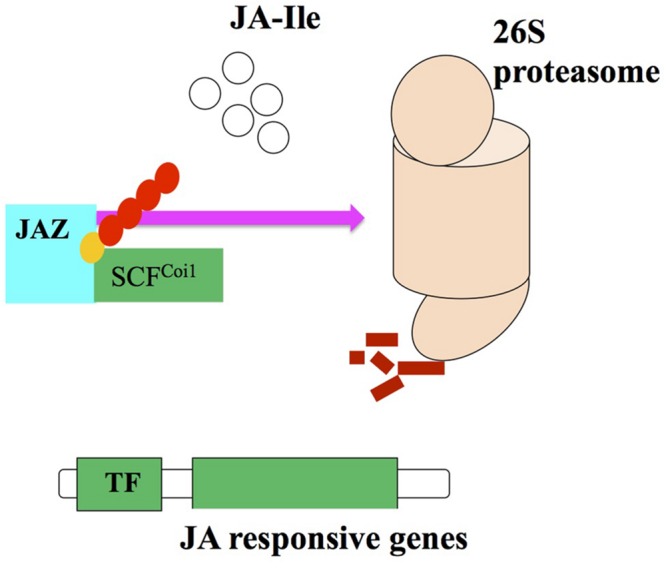
**Proteasome degredation via interaction of JA2 proteins with SCF^Coi1^ ubiquitin ligase upon JA-Ile perception and activating the JA responses (adopted from Ballare, 2011 with modifications)**.

Despite all these reports, a possible target for the Skp1/Cullin/F-boxCOI1 (SCF^Coi1^) complex, there is no any information available regarding the repressor that can provide evidences about the relationship between entire transcriptome and the SCF^Coi1^ complex regulated by JA (Browse, 2009). The identification of the SCF^Coi1^ complex target molecule in the JASs signaling transduction. Thines et al. (2007) found upregulation of 31 genes over control, after treatment of *Arabidopsis* opr3 mutant with JA. Out of 31 genes, seven possess a 28-amino acid conserved domain (ZIM domain), eight code for the proteins with unknown function so these genes were named JASMONATE ZIM DOMAIN (jaz). The transgenic plants raised from JAZ1-GUS and JAZ6-GUS and the control plant containing GUS gene only showed intense blue color. Treatment of transgenic and control plants with 100 μM JA for 1 h showed complete loss of GUS activity in transgenic plants with JAZ6-GUS and JAZ1-GUS, however, no loss in color was noticed in non-transgenic plants. Hence, it was suggested that Coi1 and JAZ proteins interaction is facilitated by JAs and consequently they help JAZ proteins to cause degradation by ubiquitination and proteasome 26S (Koo et al., 2011; **Figure 3**).

A protein JASMONATE INSENSITIVE3 (JAI3) has been identified by Chini et al. (2007) and it is included in JAZ protein family, which is recognized as a primary repressor of gene expression in the JAs signaling. JAI3 and other JAZ proteins have been reported to interact with Coi1. The JA treatment degrades proteasome 26S due to the activity of Coi1 (Chung et al., 2010). Robson et al. (2010) demonstrated that JA signaling against shade and wounding are interlinked with JAZ1 stability in *Arabidopsis*. So, it can be recommended that JAI3/JAZ3 and JAZ1 are repressors of the JA signaling. In addition, SCF^Coi1^-dependent ubiquitination is required for the activation of JA responsive genes (Chini et al., 2007; Kazan and Manners, 2008).

Generally, plant adaptation to stresses is interlinked with stimulation of cascades of molecular processes including stress perception followed by transduction of a signal, and the expression of specific genes. As a result, plant stress tolerance capacity of transgenes is improved due to regulation of the function as well as structure of cellular components (Ashraf and Akram, 2009; Tuteja et al., 2012). For example, a wound-inducible transcript *JASMONATE-ASSOCIATED1* (*JAS1*) regulated by the JA pathway was introduced into *A. thaliana* plants by Yan et al. (2007). They observed that the roots of transgenic *Arabidopsis* plants were better in growth than those of wild plants. Moreover, they confirmed this by using RNA interference by suppressing the action of JAS1 and found growth inhibition (Yan et al., 2007). In another study, effectiveness of MeJA in tuber formation in potatoes was assessed by introgression of *Arabidopsis JMT* (JA carboxyl methyltransferase) in potato plants. A significant improvement in number, size and weight was observed in transgenic potato plants due to overexpression of *JMT* mRNA levels (Sohn et al., 2011). Both JA and MeJA have shown a prominent role in the regulation of plant defense genes against different stresses. AOS converts hydroperoxide (lipoxygenase-derived fatty acid) to allene epoxide (a precursor of JA synthesis) in potato plants that led to an increase in the endogenous level of JA. Transgenic potato plants containing flax AOS cDNA had 6–12-fold increase in JA as compared to control plants. Similarly, the transgenic plants showed high expression of either drought stress- or wound-inducible genes (Harms et al., 1995). In addition, introgression of *JMT* obtained from *Arabidopsis* introduced into *Escherichia coli* catalyzed the formation of MeJA from JA. JMT RNA was identified in flowers, leaves, and rosettes of seedling plants, which indicated that JMT could respond to systemic or local signals produced in response to environmental stimuli. The transgenic plants were more resistant to *Botrytis cinerea*, a virulent fungus (Seo et al., 2001).

All these studies along with those mentioned in **Table 2** clearly indicate that a number of components/genes of JA/MeJA including its receptors have been identified, but still more need to be explored. Up till now, a variety of roles of JAs have been observed in stress tolerance (biotic/abiotic), but the complete genetic cascade of JAs involved therein is partially determined which could be explored by comparing plants enriched/deficient in JAs after subjecting them to stress or non-stress conditions.

**Table 2:** Transgenic plants over-expressing genes involved in biosynthesis and/or signaling of jasmonates.

Transgenic plants	Donor	Gene/transcript	Regulation in different plant attributes	Reference
Potato (*Solanum tuberosum* L.)	Flax (*Linum usitatissimum*)	Allene oxide synthase (*AOS*)	Transgenic plants had 6–12-fold higher level of JA than the wild plants mechanical wounding. However, transgenic plants with increased level of JA did not show changes in water state or in the expression of water stress-responsive genes	Harms et al., 1995
*Arabidopsis thaliana*	*A. thaliana*	*JASMONATE-ASSOCIATED1* (*JAS1*)	The roots of transgenic plants overexpressing a gene of unknown function were longer than those of wild-type plants	Yan et al., 2007
Soybean [*Glycine max* (L.) Merrill.]	*Brassica campestris*	*NTR1*	Transformed plants showed 2–2.5-fold higher level of MeJA, better plant height, lateral root development, but less primary root elongation as compared to the wild-type plants	Xue and Zhang, 2007
*Arabidopsis thaliana*	*Escherichia coli*	Jasmonic acid carboxyl methyltransferase (*JMT*)	Transgenic plants showed 3-fold increase inendogenous MeJAlevel but no change in JA was observed. In addition, more resistance against the virulent fungus *Botrytis cinerea* was observed in transgenic plants	Seo et al., 2001
*Panax ginseng*	*A. thaliana*	*Arabidopsis* jasmonic acid carboxyl methyltransferase (*AtJMT*)	Transgenic plants showed high expression of *PgSS1,PgSE1*, and *PgDDS* involved in ginsenoside biosynthetic pathways as well as a MeJA-responsive gene, *PgPR10-2*	Kim et al., 2012
Rice (*Oryza sativa* L.)	*A. thaliana*	*AtJMT*	Seven different genes were regulated in both Ubi1:*AtJMT* and drought-treated wild plant. Two genes, *OsSDR* and *OsJMT1* were involved in MeJA and ABA biosynthesis, respectively	Kim et al., 2009
Potato (*S. tuberosum* L.)	*A. thaliana*	*JMT*	Increased tuber yield and size in transgenic potato plants. In addition an increase in JA, MeJA and tuberonic acid (TA) levels and expression of allene oxide cyclase (*AOC*) and proteinase inhibitor II (*PINII*) genes were also observed in transgenic plants as compared to the control plants	Sohn et al., 2011
Tobacco (*Nicotianatobacum* L.)	- - - - - - - -	By silencing monogalactosyldiacylglycerol (*MGDG*)	In response to wounding, the transgenic plants produced lower levels of JA than wild-type plants. In addition, lipoxygenase (*LOX1*), *AOC*, hydroperoxidelyase (*HPL*) and proteinase inhibitor (*PI-I* and *PI-II*) were strongly diminished in transgenic plants while they were highly activated in wild-type plants on mechanical wounding	JunBin, 2009

## Crosstalk Between Jasmonates and Other Defense Signaling Pathway through MAPK Cascades

Mitogen activated protein (MAP) kinases are serine/threonine protein kinases that are involved in different intra- and extra-cellular signals. These MAP Kinases form a cascade where MAPK is phosphorylated and activated by MAPK kinase (MAPKK) followed by MAPKK kinase (MAPKKK; Ahmad et al., 2008). The involvement of MAPKs in JA or wound signaling in different plants has been reported (Meldau et al., 2012).

Seo et al. (1995) identified a wound-induced protein kinase (WIPK) in tobacco. The WIPK along with SIPK (SA-induced protein kinase) are responsible for modulating anti-herbivore secondary metabolites and other herbivory-induced phytohormones (Heinrich et al., 2011). Expression of WIPK is genetically suppressed in transgenic tobacco plants and due to this they do not show synthesis of JA on injury. According to Farmer and Ryan (1992) early wound signal enhances the phosphorylation of protein kinases and subsequently stimulates the activity of enzymes involved in JAs biosynthesis. However, JA-insensitive *A. thaliana* dwarf mutant, *mpk4* (MAPkinase4) has been observed to accumulate SA and show high expression of pathogenesis related protein (PR1) along with high resistance to *P. syringae* (Petersen et al., 2000). Plant reduction of dwarfing and no expression of PR1 have been observed in *mpk4* plants having *nahG* gene encoding SA hydrolase, that decreased SA level (Turner et al., 2002). The expression of defense related genes, *SAR* and *PR1*, indicated that *MPK4* acts as a mediator in the crosstalk between JA and SA pathways (Leon-Reyes et al., 2010).

In *Arabidopsis*, a JA-activated MAPK cascade, MAPK kinase3-MAPK6, has been identified by Takahashi et al. (2007). Menke et al. (2004) earlier showed in *Arabidopsis* that MPK6 is a component of disease resistance. JA downregulates the TF *MYC2*, which was previously found to suppress JA/ET-related genes (Lorenzo et al., 2004). Takahashi et al. (2007) demonstrated that MKK3 also regulates this cascade. According to Takahashi et al. (2007) MKK3-MPK6 cascades involved in regulation of gene expression are dependant on JA/ET. However, MPK6 controls signals like cold, pathogens, salt, and JA is presented in a model in which MPK6 functions in three different pathways (Balbi and Devoto, 2008). The MKK4/MKK5-MPK6 and MKK2-MPK6 cascades were thoroughly studied by Lorenzo et al. (2004) and they found that they significantly regulated salt and chilling stress responses and ethylene induced plant defense (PDF1.2). The MKK3-MPK6 pathway retarded JA-induced root growth as well as gene expression of Vegetative Storage Protein2 (VSP2) via MYC2, a TF which regulates ethylene pathway by suppressing PDF1.2

Various MAP kinases are activated in *Arabidopsis* during wounding like MPK4, MPK6 and MPK1/MPK2 (Ortiz-Masia et al., 2007). In the absence of wound, MPK1/MPK2 gets activated by JA. Ortiz-Masia et al. (2007) found that wound and JA induction of MPK1/2 is Coi1-dependent. In addition, H_2_O_2_ and ABA also activate MPK1/2, suggesting their role under stress conditions. Zhang et al. (2011) demonstrated that the group C (*GhMPK2*) MAPK gene isolated from cotton has a role in signaling pathway and defends the plants from pathogens and oxidative stress. Over-expression of *GhMPK2* in transgenic tobacco showed a significant resistance to viral and fungal attack due to over-expression of pathogenesis-related (PR) genes, such as PR1, PR2, PR4, and PR5 in transgenic plants (2011). García-Andrade et al. (2011) also demonstrated the role of defense-related gene (OP3) in *Arabidopsis*. Here double mutants showed callose deposition regulation by JA and ABA in response to necrotrophic fungal pathogens like *B. cinerea and Plectosphaerella cucumerina*.

Camp et al. (2000) demonstrated that MAK phosphatases (MKP) are repressors of MAPKs. MKPs have been observed to play a role in abiotic stresses and hormonal regulation in *Arabidopsis* and tobacco (Naoi and Hashimoto, 2004). Reports about the significance of MKPs in monocots are sparse. Wounding induces OSMKP1 expression in rice and it has been shown that OSMKP1 acts as a suppressor of wound responses (Balbi and Devoto, 2008).

Schweighofer et al. (2007) identified a novel stress signal regulator in *Arabidopsis*, i.e., AP2C1 which is a MAPK Ser/Thr phosphatase. Overexpression of AP2C1 makes the plants to lose their resistance potential to *Botrytis* and also causes suppression in ethylene biosynthesis (Balbi and Devoto, 2008). G-proteins are also reported to be linked with MeJA signaling (Trusov et al., 2006; Okamoto et al., 2009). Recently, it was shown that treatments of *Pisum sativum* seedlings with MeJA upregulated MPK3 and G-beta subunit of G-proteins genes which later resulted in their interaction (Bhardwaj et al., 2011). Recently, Meldau et al. (2012) observed that ir*WIPK* and LOX3 deficient *N. attenuata* plants performed better as compared to WT ones. Similarly, ir*SIPK* mutant plants, showed suppression in JA signaling and performed not well as did the WT plants. So it can be suggested that signaling mediated by *WIPK* and *SIPK* is not involved in growth promotion of *N. attenuata* plants. Both MAPKs played a differential role in regulating the plants’ growth-defense balance (Meldau et al., 2012). In addition, *Arabidopsis* MAP kinase (*mpk6*), *jar1* and *coi1* mutants plants were altered in JA signaling as well as they were not protected from fungal attack even on *N*-isobutyl decanamide exogenous application which is opposite to those of SA *eds16/sid2-1* mutants (Mendez-Bravo et al., 2011).

It has been found that MAPK kinases are important components of JA signaling and biosynthesis pathways, but NO, ROS, Ca, ABA, ethylene, and SA are also important regulators of plant fitness during JA signal transduction and synthesis. The exploration of other signaling molecules can be beneficial to examine the details of underlying molecular mechanisms of JA signal transduction.

## Conclusion and Future Perspectives

It is now clear that JAs are involved in a diversity of functions. Much progress has been made in identification and characterization of several enzymes involved in biosynthesis and metabolism of JA and its derivatives. Among the new chemicals identified, ‘arabidopsides,’ esterfied oxylipin derivatives, are of great importance. They play a crucial role in plant defense. Recent findings on the role of JA in reproduction, flower nectar secretion, G-protein signaling, and cancerous diseases have opened new vistas for future research.

In view of the above-mentioned studies it is clear that JAs play an essential role from seed germination to senescence, but there is limited information available in the literature on how these plant processes vary from species to species with JA application.

A diverse range of components/genes of JA/MeJA including its receptors have been identified (**Table 2**), but their appropriate functions still need to be explored. In addition, the complete genetic cascade of JAs involved in genetically engineered transgenic plants is partially determined which could be explored by comparing plants enriched/deficient in JAs after subjecting them to stress or non-stress conditions.

Much work is to be done in near future to find out the proper answers of the questions like action of JA metabolites, and identification of universal JA receptors etc. Complete signaling pathways involving MAPKs, CDPK, TGA, SIPK, WIPK, and WRKY TFs are yet to be studied to understand the complete mechanism of action of JA.

The most important aspect is that the extent of effectiveness of JAs like other hormones is plant species dependent. They affect the growth of different plants to a different extent. JAs play a role from germination up to senescence. However, various genes involved in growth regulation at different stages of development, are yet to be identified. How far and up to what extent these genes are involved in cross-talks also needs to be explored.

## Author Contributions

PA, SR, and AG wrote the manuscript. SAS, NAA, MA, and SG contributed in section 2 and 3 of this manuscript. They also reviewed and updated the manuscript.

## Conflict of Interest Statement

The authors declare that the research was conducted in the absence of any commercial or financial relationships that could be construed as a potential conflict of interest.
